# Early prediction of moderate-to-severe condition of inhalation-induced acute respiratory distress syndrome via interpretable machine learning

**DOI:** 10.1186/s12890-022-01963-7

**Published:** 2022-05-12

**Authors:** Junwei Wu, Chao Liu, Lixin Xie, Xiang Li, Kun Xiao, Guotong Xie, Fei Xie

**Affiliations:** 1 Library of Graduate School, Chinese People’s Liberation Army General Hospital, Beijing, 100853 China; 2 Ping An Healthcare Technology, Beijing, China; 3 College of Pulmonary and Critical Care Medicine, Chinese People’s Liberation Army General Hospital, Beijing, 100853 China; 4 Ping An Health Cloud Company Limited, Beijing, China; 5 Ping An International Smart City Technology Co., Ltd., Beijing, China; 6 Yidu Cloud Technology Inc, Beijing, China

**Keywords:** Early prediction, Etiology-specific, ARDS (acute respiratory distress syndrome), Critical care, Interpretable machine learning

## Abstract

**Background:**

Several studies have investigated the correlation between physiological parameters and the risk of acute respiratory distress syndrome (ARDS), in addition, etiology-associated heterogeneity in ARDS has become an emerging topic quite recently; however, the intersection between the two, which is early prediction of target conditions in etiology-specific ARDS, has not been well-studied. We aimed to develop and validate a machine-learning model for the early prediction of moderate-to-severe condition of inhalation-induced ARDS.

**Methods:**

Clinical expertise was applied with data-driven analysis. Using data from electronic intensive care units (retrospective derivation cohort) and the three most accessible vital signs (i.e. heart rate, temperature, and respiratory rate) together with feature engineering, we applied a random forest approach during the time window of 90 h that ended 6 h prior to the onset of moderate-to-severe respiratory failure (the ratio of partial pressure of arterial oxygen to fraction of inspired oxygen ≤ 200 mmHg).

**Results:**

The trained random forest classifier was validated using two independent validation cohorts, with an area under the curve of 0.9127 (95% confidence interval 0.8713–0.9542) and 0.9026 (95% confidence interval 0.8075–1), respectively. A Stable and Interpretable RUle Set (SIRUS) was used to extract rules from the RF to provide guidelines for clinicians. We identified several predictive factors, including resp_96h_6h_min < 9, resp_96h_6h_mean ≥ 16.1, HR_96h_6h_mean ≥ 102, and temp_96h_6h_max > 100, that could be used for predicting inhalation-induced ARDS (moderate-to-severe condition) 6 h prior to onset in critical care units. (‘xxx_96h_6h_min/mean/max’: the minimum/mean/maximum values of the xxx vital sign collected during a 90 h time window beginning 96 h prior to the onset of ARDS and ending 6 h prior to the onset from every recorded blood gas test).

**Conclusions:**

This newly established random forest‑based interpretable model shows good predictive ability for moderate-to-severe inhalation-induced ARDS and may assist clinicians in decision-making, as well as facilitate the enrolment of patients in prevention programmes to improve their outcomes.

**Supplementary Information:**

The online version contains supplementary material available at 10.1186/s12890-022-01963-7.

## Background

Acute respiratory distress syndrome (ARDS) is life-threatening and the major cause of morbidity and mortality in intensive care units (ICUs), with a mortality rate exceeding 40% [1, 2]. Despite the fact that therapies, such as ECMO, were well developed in the modern era, those did not remarkably reduce the mortality of the disease, this is because guidelines provide a uniform recommendation while neglecting the effect of case-mix in etiologies. Etiology-specific patients are more likely to respond to a given therapy [3]. Therefore, early prediction of patients with a high risk for developing moderate-to-severe ARDS and the use of prevention strategies for such patients are of great value in critical care units [4]. However, healthcare providers experience challenges in recognising ARDS patients [5], which may be due to the diversity of causes (e.g. inhalation, trauma, coronavirus disease [COVID-19]) [4]. Additionally, ARDS is rarely present at the time of hospital admission [5, 6], increasing the risk of clinicians not readily recognising, interpreting, and acting upon relevant information [7].

Because of the high mortality and difficulty in disease recognition, understanding the relationship between risk factors and ARDS is of considerable value. For example, serum zinc levels have been recommended as an appropriate biomarker for evaluating the risk of developing inhalation-induced ARDS [8]. Additionally, XGBoost, an open-source software library, has been adopted to identify ARDS based on non-invasive physiological parameters, such as heart rate and respiratory rate [9]. Alternatively, machine-learning has recently been used for the early prediction of ARDS [5]. Although ARDS prediction has attracted the attention of many researchers, previous studies have been focused on all-cause ARDS, without considering the etiology of the disease, and studies on etiology-specific ARDS prediction are lacking. In addition, it is challenging to translate machine-learning methods into clinical practice owing to the following reasons: (1) most models have not been validated using an independent test set and therefore have unknown generalisability [10]; (2) ensemble learning models (e.g. XGBoost), despite providing good prediction performance, lack transparency (interpretability); and (3) many models incorporate too many indices (e.g. laboratory blood tests), increasing their potential complexity and burden in clinical practice [5, 9–11].

To overcome the above obstacles, we focused on inhalation-induced ARDS. Smoke inhalation was one of the leading causes of pulmonary ARDS after pneumonia [3]. Owing to the nature of the injury (e.g. smoke from fires, smoke bombs), patients with inhalation-induced ARDS often present in groups, increasing pressure on clinical practitioners. Limited access to health care resources, such as low nurse-to-patient ratios, has made the situation worse; for example, it has caused delays in initiating and providing adequate treatment for deteriorating patients and higher ICU mortality [7]. Thus, this study aimed to develop a simple transparent (interpretable) model to predict early moderate-to-severe inhalation-ARDS with generalisability using data from eICUs [12], as a retrospective derivation cohort, and independently validate the model with Cohort 1 (PLAGH): patients with acute HC/Zinc chloride smoke inhalation lung injury who were admitted to the respiratory department at People's Liberation Army General Hospital (PLAGH) in 2014; Cohort 2: freely available critical care data from the Medical Information Mart for Intensive Care III (MIMIC-III; www.mimic.physionet.org) [13]. Our model is able to reliably predict the onset of moderate-to-severe inhalation-induced ARDS 6 h prior to onset.

## Methods

This study used a random forest approach for the early prediction of inhalation-induced ARDS. To develop the model, we used a retrospective observational cohort, obtained from the eICU Collaborative Research Database that consists of inpatient ICU encounters at 32 de-identified medical centres between 2014 and 2015. Fifteen patients from respiratory department at PLAGH and nine patient encounters from the MIMIC-III database were used as a validation cohort. eICU and MIMIC-III databases are publicly accessible, and the MIMIC-III publication states that ‘the project was approved by the Institutional Review Boards of Beth Israel Deaconess Medical Center (Boston, MA) and the Massachusetts Institute of Technology (Cambridge, MA)’. Requirement for individual patient consent was waived because the project did not impact clinical care and all protected health information was deidentified. This study is reported following the STrengthening the Reporting of OBservational studies in Epidemiology (STROBE) guidelines. Additional file 1 shows the completed STROBE checklist.

**Fig. 1:**
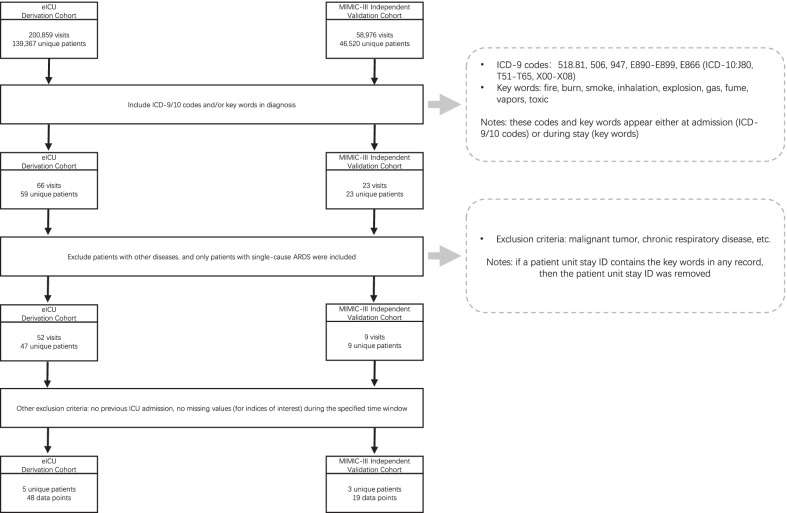
The patient selection process (including the number of patients after each selection procedure)

Trained investigators extracted data from electronic patient medical records from the two public cohorts. Selection of patients was based on ICD-9/10 codes and diagnostic key words. Patients with previous ICU admission were excluded. Each patient was cared for at a single medical centre. We applied additional inclusion criteria to focus the scope of our study. Patient stays that did not involve at least one recorded measurement of the variables were excluded [5, 11]. In addition, we included only patient stays with a duration within a specified window (see “Predictor variables” section for details). The final derivation cohort (i.e. eICU) consisted of five unique patients corresponding to 48 unique observations. The final validation cohort (i.e. PLAGH, MIMIC-III) comprised 15 unique patients (177 observations), and three unique patients corresponding to 19 observations. The characteristics of both cohorts are presented in Table 1. Figure 1 outlines the patient selection process and details the number of patients after each selection procedure.

**Table 1:** Characteristics of the final cohorts

	Derivation cohort	Validation cohort		*p* value
	eICU	PLAGH	MIMIC-III	
# Data points (subjects N)	48 (5)	177 (15)	19 (3)	–
# Positive labels (total)	26 (48)	79 (177)	3 (19)	< 0.05
Age, years	42.2 (31.0–57.6)	20.7 (18.7–23.3)	63.3 (48.6–84.6)	< 0.05
Females	1 (5)	0 (15)	0 (3)	0.15
Hospital discharge status–death	2 (5)	1 (15)	0 (3)	0.12
Ethnicity, N (%)				
White	4 (80%)	0	2 (66.67%)	
Hispanic	1 (20%)	0	0	< 0.05
Asian	0	15 (100%)	0	
Others (unknown)	0	0	1 (33.33%)	
Length-of-stay (LOS) hours (difference between admission and discharge)	321.8	214.5, 262.6, 1048	85.5	
	44.1	201.8, 783.0, 238.6	181.5	
	92.0	231.0, 258.3, 282.2	91.0	0.31
	849.5	1048, 214.6, 273.8		
	4.2	158.8, 272.8, 475.1		

Data are presented as mean (95% range) or N (proportion), unless otherwise stated

### Outcome variables

The primary outcome of interest in this study was the ratio of partial pressure of arterial oxygen to fraction of inspired oxygen (PaO_2_/FiO_2_ ratio) 6 h since investigation. ARDS was diagnosed according to the Berlin definition established in 2012 [14]. Depending on the severity of lung failure according to the PaO_2_/FiO_2_ ratio, ARDS is currently classified as mild (200 < PaO_2_/FiO_2_ ≤ 300), moderate (100 < PaO_2_/FiO_2_ ≤ 200), or severe (PaO_2_/FiO_2_ ≤ 100) [14, 15]. In the model development, the numeric ratio was converted to a binary outcome: presence of moderate or severe ARDS (PaO_2_/FiO_2_ ≤ 200) versus mild or better ARDS (PaO_2_/FiO_2_ > 200).

### Predictor variables

For model development, the predictive power of three primary vital signs (body temperature, pulse (heart rate [HR]), and breathing rate (respiratory rate)), were evaluated. These were selected as predictor variables because they are the most accessible, non-invasive physiological parameters that are continually monitored. In addition, measurements for these vital signs generated in different platforms are easily compared as they are not affected by external factors or obtained by means of different medical devices. Thus, if a correlation between the vital signs and ARDS could be established, it would allow a prediction with simplicity and generalisability.

**Fig. 2:**
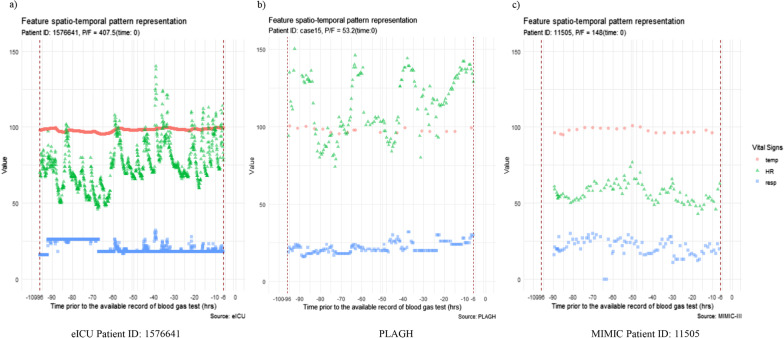
The feature’s spatiotemporal pattern representation for three representative patients from each cohort

Feature engineering was performed before features were included in the random forest analysis. We adopted a “time-phased” strategy that calculated the minimum, maximum, and mean values of the three vital signs collected during a 90 h time window beginning 96 h prior to the onset of ARDS and ending 6 h prior to the onset from every recorded blood gas test (by which the PaO_2_/FiO_2_ ratio could be calculated) [16]. The feature’s spatiotemporal pattern representation is shown in Fig. 2 for three representative patients from each cohort.

### Model development

We adopted a random forest classifier, an ensemble learning method for classification, to predict the binary diagnosis outcome of ARDS. In the random forest analysis, each tree was constructed using a different bootstrap sample from the original training data [17]. The parameter “Number of trees” was set to 5,000 to ensure that every input row would be predicted at least a few times; all other parameters were set to default. During the initial model training process, nine predictor variables during the 90-h time window were included: minimum, maximum, and mean HR; minimum, maximum, and mean respiratory rate; and minimum, maximum, and mean temperature. The importance of each variable was ranked according to the mean decrease in Gini values. The top four features (i.e. resp_96h_6h_mean, HR_96h_6h_mean, resp_96h_6h_min, and temp_96h_6h_max) were finally selected because their importance, measured according to the mean decrease in Gini value, was higher than the average mean decreases in Gini for all candidate features. In this way, we could halve the number of features in the model for a small cost in accuracy.

Seven prediction models, including the random forest, were developed for comparison: multivariate logistics regression (MLR), Lasso regression, random forest and XGBoost were developed for both four-variable and all-variable sets as appropriate. Optimal parameters of ML algorithms (e.g., XGBoost) were obtained through cross validation on training set (i.e., eICU). Model performances were compared on two independent validation sets (i.e. PLAGH, MIMIC).

### Statistical analysis

Cohort characteristics were expressed as mean (range) and proportions (n, %), as dictated by the data type. We used the analysis of variance (ANOVA) to compare means of continuous variables of more than two groups, and χ^2^ test to compare the frequencies of categorical variables between groups. Gini-based feature selection is implemented in the randomForest R package [18].

Receiver operating characteristic (ROC) curves were generated to quantify the predictive accuracy of the models, and the area under the curve (AUC) was used to assess the discriminatory ability of the models. Cut-off threshold for optimal point was determined by performing ROC curve analysis in the retrospective cohort [6].

Data were analysed using R V.4.0.1. Two-sided *p* values < 0.05 were considered statistically significant.

## Results

### Performance on independent test set

**Fig. 3:**
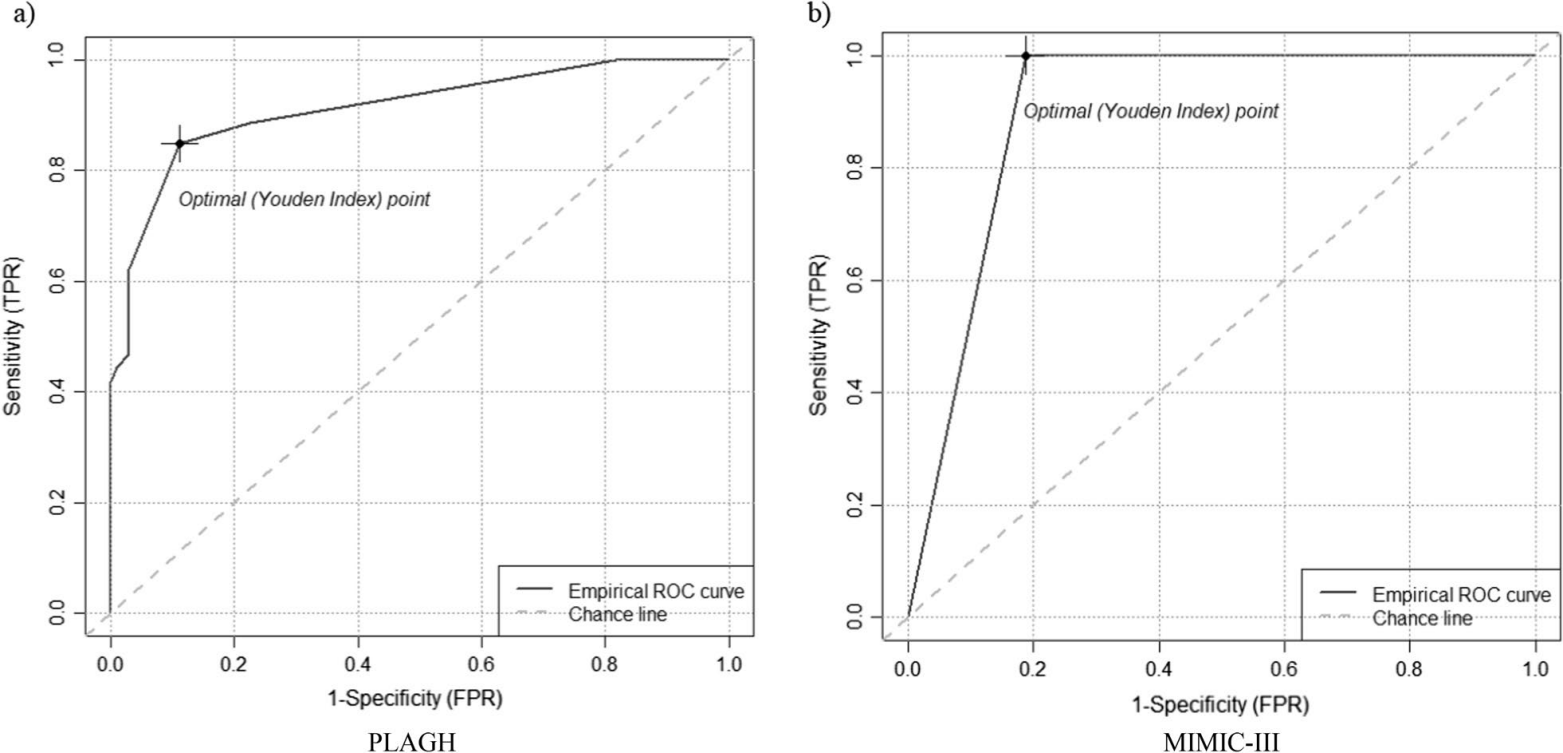
Receiver characteristic operating (ROC) curves of the application of the model on the independent validation sets (PLAGH, MIMIC III

The binary ARDS diagnosis prediction performance by our final model (i.e. RF with selected variables) is summarised using a ROC curve in Fig. 3. Model performance comparison was shown in Additional file 2: Table S1. The random forest with the selected four features performed better than other models (e.g., MLR, Lasso, XGB) The final model (i.e., Four-Var RF) demonstrated an area under the ROC curve (AUROC) of 0.9127 (95% confidence interval [CI] 0.8713–0.9542) and 0.9026 (95% confidence interval [CI] 0.8075–1) for early ARDS prediction with the independent validation sets (PLAGH, MIMIC-III) 6 h prior to the onset of moderate or severe ARDS. The corresponding ROC curve is shown in Fig. 3. The final model was internally validated and the bootstrap estimate of AUC was 0.8391 (500 times).

We also measured the prediction performance of the model using other evaluation metrics (i.e. sensitivity, specificity, and accuracy. The results are presented in Table 2.

**Table 2:** Prediction performance of the model using different evaluation metrics

Cut-off threshold	PLAGH					MIMIC-III				
	Accuracy	Sensitivity	Specificity	Positive predictive value	Negative predictive value	Accuracy	Sensitivity	Specificity	Positive predictive value	Negative predictive value
0.1	0.4463	1	0	0.4463	NA	0.1579	1	0	0.1579	NA
0.2	0.4463	1	0	0.4463	NA	0.1579	1	0	0.1579	NA
0.3	0.4463	1	0	0.4463	NA	0.1579	1	0	0.1579	NA
0.4	0.8249	0.8861	0.7755	0.7609	0.8941	0.5789	1	0.5	0.2727	1
0.5	0.7458	0.443	0.9898	0.9722	0.6879	0.5789	1	0.5	0.2727	1
0.6	0.7401	0.4177	1	1	0.6806	0.6316	1	0.5625	0.3	1
0.7	0.5537	0	1	NA	0.5537	0.8421	0	1	NA	0.8421
0.8	0.5537	0	1	NA	0.5537	0.8421	0	1	NA	0.8421
0.9	0.5537	0	1	NA	0.5537	0.8421	0	1	NA	0.8421

### Model interpretation

The lack of interpretability of the ensemble learning methods is a strong limitation for applications involving clinical decision-making. To overcome this issue, we employed an interpretable random forest model, Stable and Interpretable RUle Set (SIRUS) [19], as implemented in the R package SIRUS [20], to extract rules from a tree ensemble based on their frequency of appearance. As stated in the original method article “the most frequent rules, which represent robust and strong patterns in the data, are ultimately linearly combined to form predictions” [21]. SIRUS outputs a simple set of 10 rules (with all parameters set to default). To generate the prediction for each specific instance, SIRUS considers whether the conditions for each rule are satisfied to assign one of the two possible likelihood output values. In the next step, the 10-rule outputs were averaged to provide the predicted probability of the onset of moderate or severe ARDS. The 10-rule set is presented in Table 3. Overall, the rules, generated by the interpretable model, provide transparent and concise information regarding how the prediction is made, thus overcoming the aforementioned challenges hindering translational research of ARDS.

**Table 3:** Rules for calculating the likelihood of moderate to severe ARDS developing in 6 h

No.	Conditions	Satisfied	Not satisfied
1	resp_96h_6h_min < 9	0.864	0.269
2	resp_96h_6h_mean < 16.1	0	0.684
3	HR_96h_6h_mean < 102	0.447	0.9
4	temp_96h_6h_max < 100	0.292	0.792
5	temp_96h_6h_max < 100	0.222	0.733
6	resp_96h_6h_mean < 19	0.211	0.759
7	resp_96h_6h_mean ≥ 16.1 & resp_96h_6h_min ≥ 9	0.412	0.613
8	HR_96h_6h_mean < 84.3	0.333	0.636
9	resp_96h_6h_mean ≥ 16.9 & resp_96h_6h_min < 9	0.905	0.259
10	HR_96h_6h_mean < 110	0.488	1

## Discussion

In this study, we developed a machine-learning model for the early prediction of inhalation-induced ARDS (moderate-to-severe condition) at 6 h prior to onset in critical care units. The model demonstrated good prediction performance and clinical interpretability on eICU data. Specifically, the model achieved a high AUROC with two completely independent external validation set (i.e. PLAGH, MIMIC-III). All features used in the model were derived from clinical variables that are routinely/continuously collected in ICUs, which can be simply and non-invasively obtained. This clinically applicable model can be easily applied to ICU patients to assist clinical decision-making and thus, holds great potential for improving patient outcomes.

Importantly, the model provided a convenient way of calculating the likelihood of the target event, i.e. the development of inhalation-induced ARDS (moderate-to-severe condition). To the best of our knowledge, no previous study has used the SIRUS in a clinical setting. The model improved prediction performance based on the random forest approach while maintaining transparency. More precisely, the rule set and how each of the rules contributed to the prediction was clearly shown. Instead of a decision-making path (where rules work interactively), those rules work independently towards a probability, making mechanistic exploration and/or explanation more realistic. It is noteworthy that we conducted experiments with both linear (e.g., MLR, Lasso) and non-linear (e.g., RF, XGBoost) algorithms, and chosen RF based on a data-driven approach, because the discrimination ability of RF is better than other algorithms (the comparison of the prediction performance of different ML algorithms in validation sets was in Additional file 2: Table S1).

To generate the predicted likelihood of the presence of moderate to severe acute respiratory distress syndrome (ARDS), the model considers whether the conditions for each rule are satisfied to assign one of the two possible output values. For example: resp_96h_6h_min = 5:0 (minimum value of respiratory rate of all available respiratory rate measured during 96–6 h prior to the target time point), then the first rule is satisfied, returning p (1) = 0.864. Next, the 10 rule outputs are averaged to provide the predicted probability of ARDS (moderate or severe) onset.

There are several vital signs typically examined by medical professionals, such as temperature, HR, and respiratory rate. Additionally, although blood pressure is not considered a vital sign, it is often measured alongside the vital signs. However, the three vital signs are generally more accessible and accurate than blood pressure, which is more prone to influence from external factors [22]. Moreover, model simplicity can lead to higher feasibility of implementation, particularly in low-income countries that lack ICU capacity. Furthermore, because of infrequent vital sign monitoring and a lack of standardised management practices in resource-limited settings, basic monitoring measures need to be better used [23]. Thus, we included only three simple, non-invasive, routinely monitored vital signs in our model.

The risk factors identified in our study are consistent with those from previous studies and were validated using an independent validation set. In 2020, Liu et al. compared the most commonly used early warning scoring systems for hospitalised patients with and without infection at risk for in-hospital mortality and transfer to the ICU and found that the National Early Warning Score (NEWS) exhibited the highest discrimination for mortality (followed by the Modified Early Warning Score-MEWS) [24]. The main findings of the present study agreed with the role of the NEWS [25]. For example, a respiratory rate ≤ 9 breaths/min increases the risk of developing critical or moderate-to-severe ARDS according to both the NEWS and our model. To further compare the discrimination capability of our model to that of commonly used risk scoring systems, we calculated MEWS and Systemic Inflammatory Response Syndrome (SIRS) for each data point of the included patients in the 90-h time window and the real time of the recorded blood gas test both. Discrimination capability of the scores based on AUCs was compared in Additional file 2: Table S2. Our model had a better performance in early warning of moderate to severe ARDS given the 90-h window performance and it also had a better reproducibility and generalizability as its AUC is stable on two independent validation sets both.

Rather than providing a binary outcome, our model is able to calculate the likelihood of developing moderate-to-severe ARDS. This was achieved by aggregating the vital sign measurements collected during hospital stay to yield the score used in our model, allowing the output to reflect risk for the target event. In addition, our prediction leaves a definite time window prior to the event, making potential intervention realistic. Such time-phase determination is suitable for clinical practice as clinical practitioners generally prefer having a suitable time window that is neither too far nor too near to onset, in order to leave sufficient time for intervention. The exact time window for feature engineering can be treated as a hyperparameter, which may vary slightly across different cohorts (e.g. patient characteristics) [16]. However, parameter tuning was outside the scope of this study.

In comparison with other similar studies using machine-learning models for the early prediction of ARDS, our model showed improved prediction performance. The prediction performance measured using the AUROC ranged from 0.75 to 0.87 in previous studies [5, 26, 27], whereas our model achieved an AUROC of being greater than 0.90 with two an independent validation sets. In addition, the predictors used in our model were further simplified [5]. A potential explanation may be that the retrospective cohort included in this study were etiology-specific ARDS patients (ARDS patients with inhalation injury). The improved modelling performance suggests that the etiology of ARDS could be used to identify a more homogeneous subset of ARDS for prediction enrichment which is an important component of etiology-associated heterogeneity in ARDS [3].

Although the prediction ability of the model was validated using two completely unrelated data sets, our study had some limitations. Similar to the limitations in previous data challenges “the analysis was performed in a retrospective setting. The generalisability and stability of the proposed model need to be evaluated systematically in prospective settings” [16]. Moreover, we included first admissions only, which might be a potential source of selection bias. We also acknowledge the limitation that the sample size was small. In order to overcome the issue, we extended the limited number of patients to hundreds of data points at the first place to ensure the credibility and power of model training, model validation and model performance evaluation, for example, the derivation set contained 48 data points with each class label being balanced; Secondly, the patients from different cohorts (i.e., eICU, MIMIC, PLAGH) had diversity in patient profiles, the model developed and validated on cohort with diversity had reproducibility and generalizability to new patients thereby; Last but not least, our final model was validated internally (e.g., bootstrap, LOOCV) and on two independent validation cohorts which were geographically distinct and demographically distinct from the derivation cohort. We believe that studies with a small number of subjects can quickly address research questions in a relatively short space of time; thus, a more efficient allocation of resources (e.g. subjects, time, financial costs) will be achieved by first testing a new research hypothesis in a small number of subjects [28].

## Conclusions

In this study, an interpretable machine-learning model with key features (derived from three non-invasively measured vital signs) was successfully established for predicting moderate-to-severe condition of inhalation-induced ARDS 6 h prior to onset. The prediction model is intended to improve communication between nursing staff and junior doctors and “flag” patients who need to be given immediate priority [29]. Inhalation-induced ARDS typically affects individuals such as fire fighters and soldiers, who are often in otherwise good condition without chronic diseases, and providing critical care expertise at an early stage is extremely important for improving patient outcomes.

In future research, we hope to include more patients, while expanding to other etiology-specific ARDS, such as COVID-19, as the current ARDS predictions for patients with COVID-19 are either risk factor analysis [30], real-time risk scoring systems [31], and/or utilising relatively complex predictors, such as the neutrophil-to-lymphocyte ratio [32]. We hope to accomplish this aim by integrating this early prediction model in our own ICU risk management system to evaluate its effectiveness.

In conclusion, as clinical instances accumulate and clinical records become more comprehensive, this study forms a basis for evaluating the effectiveness of personalised intervention (e.g. vital sign-directed therapy) [33]. etiology-specific medicine is a critical component of precision, personalised healthcare and will be the core of effective care in the anticipatable future.

## Supplementary Information

**Additional file 1.**Reporting items for observational research checklist - the STROBE Statement.

**Additional file 2.** Prediction performances.

## Data Availability

The data that support the findings of this study are available from Chinese PLA General Hospital, but restrictions apply to the availability of these data, which were used under license for the current study, and so are not publicly available. Data are however available from the authors upon reasonable request and with permission of Chinese PLA General Hospital.

## Abbreviations

ARDS: Acute respiratory distress syndrome
AUC: Area under the curve
CI: Confidence interval
HR: Heart rate
ICUs: Intensive care units
MEWS: Modified Early Warning Score
MIMIC-III: Medical Information Mart for Intensive Care III
NEWS: National Early Warning Score
SIRS: Systemic Inflammatory Response Syndrome
RF: Random forest
ROC: Receiver operating characteristic
SIRUS: Stable and Interpretable Rule Set
