# Supplementary material for: Early prediction of moderate-to-severe condition of inhalation-induced acute respiratory distress syndrome via interpretable machine learning
Source: BMC Pulm Med. 2022 May 12;22:193. doi: 10.1186/s12890-022-01963-7 (PMC9098141; doi:10.1186/s12890-022-01963-7)
Supplement: Supplementary file 2 — Additional file 2. Prediction performances. [file 12890_2022_1963_MOESM2_ESM.docx]

**eTable 1** Prediction performance of different ML algorithms on two independent validation sets.

| **No.** | **Model Category** | **ML Algorithm** | **Features** | **Model performance (AUC) on independent validation set 1 - MIMIC** | **Model performance (AUC) on independent validation set 2 - PLAGH** | **Notes** |
| --- | --- | --- | --- | --- | --- | --- |
| 1 | Linear | MLR | Four-variable set | 0.7708  [0.5592, 0.9825] | 0.9256  [0.8906, 0.9606] |  |
| 2 |  |  | All-variable set | 0.5  [0.247, 0.753] | 0.8994  [0.8507, 0.948] |  |
| 3 |  | Lasso | All-variable set | 0.5417  [0.2933, 0.79] | 0.9259  [0.8899, 0.9618] |  |
| 4 | Non-linear | Random forest (SIRUS) | Four-variable set | **0.9062**  **[0.8075,1]** | **0.9127**  **[0.8713, 0.9542]** | ***** |
| 5 |  |  | All-variable set | 0.9062  [0.8075,1] | 0.9101  [0.8672, 0.953] |  |
| 6 |  | XGBoost | Four-variable set | 0.9062  [0.8075,1] | 0.6983  [0.6157, 0.781] |  |
| 7 |  |  | All-variable set | 0.6563  [0.4201, 0.8924] | 0.5881  [0.5001, 0.676] |  |

**eTable 2** Prediction performance of MEWS and SIRS on two independent validation sets.

| **Scoring System** | **Time** | **Model performance (AUC) on independent validation set 1 - MIMIC** | **Model performance (AUC) on independent validation set 2 - PLAGH** |
| --- | --- | --- | --- |
| MEWS | 90-h time window | 0.0729  [0,0.2218] | 0.8895  [0.8363, 0.9427] |
|  | Real time | 0.2188  [0.219,0.4156] | 0.9471  [0.9094, 0.9848] |
| SIRS | 90-h time window | 0.3438  [0.1962, 0.4913] | 0.7964  [0.7366, 0.8563] |
|  | Real time | 0.4792  [0.1691, 0.7892] | 0.8378  [0.7702, 0.9055] |
